# Exploring the lived experience of patients and families who speak language other than English (LOE) for healthcare: developing a qualitative study

**DOI:** 10.1186/s40900-023-00465-y

**Published:** 2023-07-10

**Authors:** Victor Do, Francine Buchanan, Peter Gill, David Nicholas, Gita Wahi, Zia Bismilla, Maitreya Coffey, Kim Zhou, Ann Bayliss, Presanna Selliah, Karen Sappleton, Sanjay Mahant

**Affiliations:** 1 Temerty Faculty of Medicine, University of Toronto, Toronto, ON Canada; 2 Institute for Health Policy, Management and Evaluation, Dalla Lana School of Public Health, University of Toronto, Toronto, ON Canada; 3 Child Health Evaluative Sciences, Research Institute, Hospital for Sick Children, Toronto, ON Canada; 4 Department of Pediatrics, University of Toronto, Toronto, ON Canada; 5 Faculty of Social Work, University of Calgary, Alberta, Canada; 6 Division of General Pediatrics, Department of Pediatrics, McMaster University and McMaster Children’s Hospital, Hamilton, ON Canada; 7 Department of Pediatrics, North York General Hospital, Toronto, ON Canada; 8 Children’s Health Division, Trillium Health Partners, Mississauga, ON Canada; 9 William Osler Health System, Brampton, ON Canada; 10 Centre for Innovation and Excellence in Child and Family-Centred Care, Hospital for Sick Children, Toronto, ON Canada

## Abstract

**Background:**

Patients who use Languages other than English (LOE) for healthcare communication in an English-dominant region are at increased risk for experiencing adverse events and worse health outcomes in healthcare settings, including in pediatric hospitals. Despite the knowledge that individuals who speak LOE have worse health outcomes, they are often excluded from research studies on the basis of language and there is a paucity of data on ways to address these known disparities. Our work aims to address this gap by generating knowledge to improve health outcomes for children with illness and their families with LEP.

**Body:**

We describe an approach to developing a study with individuals marginalized due to using LOE for healthcare communication, specifically using semi-structured qualitative interviews. The premise of this study is participatory research—our overall goal with this systematic inquiry is to, in collaboration with patients and families with LOE, set an agenda for creating actionable change to address the health information disparities these patients and families experience. In this paper we describe our overarching study design principles, a collaboration framework in working with different stakeholders and note important considerations for study design and execution.

**Conclusions:**

We have a significant opportunity to improve our engagement with marginalized populations. We also need to develop approaches to including patients and families with LOE in our research given the health disparities they experience. Further, understanding lived experience is critical to advancing efforts to address these well-known health disparities. Our process to develop a qualitative study protocol can serve as an example for engaging this patient population and can serve as a starting point for other groups who wish to develop similar research in this area.

**Plain English Summary:**

Providing high-quality care that meets the needs of marginalized and vulnerable populations is important to achieving an equitable, high-quality health care system. Children and families who use a Language other than English (LOE) in English dominant regions for healthcare have worse health outcomes including a significantly increased risk of experiencing adverse events, longer lengths of stay in hospital settings, and receiving more unnecessary tests and investigations. Despite this, these individuals are often excluded from research studies and the field of participatory research has yet to meaningfully involve them. This paper aims to describe an approach to conducting research with a marginalized population of children and families due to using a LOE. We detail protocol development for a qualitative study exploring the lived experiences of patients and families who use a LOE during hospitalization. We aim to share considerations when conducting research within this population of families with LOE. We highlight learning applied from the field of patient-partner and child and family-centred research and note specific considerations for those with LOE. Developing strong partnerships and adopting a common set of research principles and collaborative framework underlies our approach and initial learnings, which we hope spark additional work in this area.

## Background

Despite recognition that social determinants are critical to health outcomes, many groups, including those with lower incomes, racial/ethnic minorities, LGBTQ2S + identifying individuals and other historically marginalized groups, continue to have suboptimal access to healthcare and worse health outcomes [1–3]. There is an ongoing need for research that incorporates patient and stakeholder engagement in developing, testing, and disseminating interventions to improve outcomes for patient populations that have been marginalized and excluded from health research.

We will refer to patients and families that do not communicate in English primarily as using a language(s) other than English (LOE) for healthcare communication in this manuscript, recognizing a number of terms are currently used.

Canadian census data indicates 12.7% of Canadians predominantly speak a language other than French or English at home [7]. Over 24% of Canadians report a mother tongue other than English or French [8]. In Toronto, a 2018 report estimated that 130,000 Torontonians did not speak any English [9]. At the Hospital for Sick Children, a free-standing children’s hospital in Toronto, Interpreter Services logged over 25 000 interpreted clinical interactions in 2020.

Patients who use a LOE in an English-dominant region are at increased risk for experiencing adverse events in healthcare settings [10, 11]. Communication issues contribute to and even directly cause serious medical errors [12]. Language barriers magnify these issues. A multicenter study by Khan et al. concluded that hospitalized children of parents with low comfort with English had twice the likelihood of experiencing harm due to improper medical care [13].

Despite the knowledge that individuals who use LOE have worse health outcomes, they are often excluded from research studies. The principle of justice, discussed in The Belmont Report, requires fair procedures and outcomes in the selection of subjects [14]. While acknowledging that language barriers can pose unique challenges, given the significant proportion of patients and families who self-report speaking English less than “very well,” this sub-population must be included in research studies.

Communication with patients and families was ranked as the second most important research question in a James Lind Alliance priority study of unanswered research questions in pediatric hospital care [13]. Studies exploring healthcare provider perceptions of interpreter use in caring for patients with LOE are common. However, studies that explore the lived experience and perspectives of patients and families hospitalized in pediatric inpatient settings who speak LOE are lacking. Studies that have been undertaken have focused mainly on single populations (e.g. Spanish-speaking in the US) and generally consider a more limited view of the hospital experience [15, 16]. Lived experience refers to the collection of experiences of an individual and the knowledge they gain from these experiences [17]. Our literature review identified a significant gap in studies on the health outcomes of pediatric patients with LOE in Canada. In particular, community based participatory research [18, 19], where researchers and community members collaborate in an equitable manner to explore and address disparities are lacking. Important questions have been minimally explored within the LOE population. Taking a community based, participatory approach through semi-structured qualitative interviews can yield critical information in helping us to address the health disparities currently experienced by patients who speak LOE. It is critical that we share best practices in how to best engage and develop study methodologies for this population.

This paper aims to discuss an approach to conducting community-based participatory research with a marginalized population of children and families due to using a LOE for healthcare communication. We detail protocol development for a qualitative semi-structured interview study exploring the lived experiences of patients and families with LOE during hospitalization through which we highlight specific principles of participatory research to consider when engaging this population. Empiric results are not detailed as this paper focuses on methodology. Given the significant efforts and careful undertaking required to develop a meaningful participatory interview study with this patient population, we strive to provide detailed insights about our process. The focus of this paper will not be on the fine details of the qualitative study protocol we have developed.

### Study design/methodology

The personal experiences of our author group as providers and family members motivated us to dedicate efforts to improving the experience and health outcomes of patients and families who use LOE for healthcare communication. The premise of our efforts was to develop a community-based participatory research study with an overall goal of systemic inquiry in collaboration with patients and families who use LOE to create an actionable agenda to eliminate the well-documented health disparities they experience.

### Study context

It is important to set the context of our study setting, which provides insights into our methodological decisions. Our study team comes from two academic and three community hospitals in southwestern Ontario, Canada. This research is conducted within the Canadian Pediatric Inpatient Research Network (PIRN, www.pirncanada.com). PIRN includes Ontario pediatric and large community hospitals. Its mission is to improve the evidence base and outcomes for hospitalized children in general pediatric settings. Canada has two official languages, English and French, though, in Ontario, most hospitals conduct their operations primarily in English. Hospitals are funded by the provincial government and patients' healthcare is covered through a provincial universal health insurance plan.

### Developing the team and considering important stakeholders

Community-based participatory research requires the right team members and stakeholders to be engaged. We took significant efforts to ensure a comprehensive, cohesive team. Our entire study team which includes patient safety experts, hospital and nursing leadership, hospital pediatricians, social workers, qualitative researchers, medical educators and patient partners, is designed to help us ensure success in meeting our study objectives. Table 1 outlines the study team roles in more detail.

**Table 1:** Important study team members and the roles and expertise they contribute to the study

Study team member	Role in study/expertise
Patient and family engagement coordinator	Critical in building connections with patients and families including connecting with patient partners Provides insight in patient and family centered research design
Centre for innovation and excellence in child and family-centred care (CIECFCC) lead	In our institution, this office oversees Interpreter services. It is critical to partner with Interpreter services on studies where they are highly involved This office also oversees patient relations. We recommend their involvement especially in studies that focus on lived experience. They can provide significant expertise in supporting families in these discussions. A pre-existing relationship with this office prior to undertaking the interviews can be helpful if/when families raise significant concerns regarding their experience
Multidisciplinary members of study team	Our study team includes physicians, social work, nursing leadership. The lived experience of a patient/family includes interactions with the multi-D team and thus a multi-D research team enriches the overall study methodology, analysis and ability to operationalize study findings
Strategic patient partner	A patient partner does more than provide a “patient perspective”. In the case of our study, they are a study team member. They provide unique insights that are critical to developing a study that is family-centered

### Overarching study design principles

Firstly, the core study team started by developing overarching principles which were agreed upon through iterative discussion after literature review and through stakeholder/study member engagement. Setting initial study design principles are critical as they serve to ground the study team in decision-making during protocol development, execution and analysis. Community based participatory research principles and core concepts of equity, diversity and inclusivity are foundational to our study design principles.

1. Patients and families are the knowledge experts – the research team’s role is to seek this knowledge and earn the trust to utilize it to bring about meaningful changes.
2. Family-Centered –When there are multiple ways to approach an aspect of the study, we ask, what is best for the family?
3. Partnership-Focused – Successfully developing a study with marginalized populations involves meaningful partnerships with stakeholders who have built relationships. Consider what the study team can offer to support the individuals we seek to partner with.
4. Adaptable – We must be open to new ways of approaching issues for inclusive and optimal impact. Traditional research approaches may need to be adapted to maximize partnerships and engagement.
5. Equity-Seeking- Our overarching goal is health equity, in addition to working to advance the health outcomes of patients and families with LOE.

### Collaborative framework

We also developed a collaboration framework to direct how we would engage with different stakeholder groups given our partnership focus. This was a critical step for us so that we could ground our interactions and ensure the study team was all on the same page with respect to our collaborative approach. These were created after initial consultation with the Research Family Advisory Committee, literature review and generative discussion amongst the study investigators. The following governs our interactions with collaborators.

1. Our partners/collaborators are the experts. We will approach all groups first to learn and then ask how we can support their efforts toward improving health equity.
2. All partners/collaborators are acknowledged and compensated for their time and efforts.
3. Respect forms the foundation of all collaborations and partnerships.
4. Equity, Diversity, Inclusivity centre our interactions.

Partnerships are initiated by reaching out to potential stakeholders and presenting overarching goals and objectives of the research, noting the opportunity for adjustments to be made in collaboration. Our relationships begin with a readiness assessment and then the groups thoughtfully consider the most appropriate engagement methods. As a study team it is important to ask for the opportunity to listen, learn, and co-develop solutions to challenges around study design and execution. It is also beneficial to ask all partners if there are others that they would suggest for collaboration and partnership.

### Approach to study design

A thoughtful, strategic, and thorough approach to study planning is critical for all community-based participatory research. In particular when working with marginalized populations and specifically in our case families who use LOE. We undertook these steps to prepare for our study. We suggest that these are important steps to develop a methodologically sound, community-based participatory study with patients and families that use LOE for healthcare communication.

A. Establishing strong communication, and addressing language barriers:

A critical issue to address is the language barriers inherent to working with patients and families who use LOE. Partnering directly with Interpreter services at our institution was an effective first step in ensuring all interactions with patients and families are with professional interpreters. It also enhances the overall study design. For our study, we partnered with the Centre for Innovation and Excellence in Child and Family-Centred Care (CIECFCC), which also oversees Interpreter Services at the Hospital for Sick Children, to ensure that we could co-design study aims and methods. Our collaboration involves regular meetings, ensuring that all methodological decisions are agreed upon, communicated, and aligned with what interpreter services can support. We provide opportunities for interpreters to be further involved in the study, including sharing insights and feedback. As we explore the lived hospital experience, families should be connected to hospital resources if they have systemic or individual care concerns that they wish to raise (beyond the research). We developed a process with the CIECFCC to pass on any concerns raised by a family. This involves direct connections with the CIECFCC leadership. While we note that the research interviews are confidential, in all instances, we offer families the opportunity to discuss any issues with CEICFCC.

1. Patient and Family Engagement in Study Design:

To obtain meaningful patient and family input and engagement in the study, our team engaged, the Patient and Family Engagement Coordinator at SickKids who has lived experience as a parent of a child with medical complexity. The Research Family Advisory Council (RFAC) at Sick Kids Hospital also played a significant role in study development. RFAC consists of patient and family representatives, research coordinators, clinician-scientists, and administrators [21]. The committee provided meaningful study input during an engagement session, raising essential points on objectives, recruitment, methodology, and the interview guide. Further, our study team engaged a patient partner compensated for their time and expertise. They bring a unique perspective as an individual who navigated the healthcare system as a child/youth due to their sibling having significant medical complexity and their family having using LOE for healthcare discussions. We listen attentively to study partners' lived experience and expertise and our study protocol is adjusted as a result. Patient and family engagement will continue to be core to our study as we recruit, interview, and analyze our data.

3 Research Ethics:

Research ethics processes are developed to protect vulnerable individuals and ensure that research teams undertake their studies in a manner that minimizes potential harm. A qualitative exploration of lived experience is a low-risk study, and we wanted to balance the burden of associated consent processes and not erect additional barriers to participation. In reviewing work in this area, we noted that Resnik and Jones wrote that US “federal and international guidance concerning this topic is insufficient, and there is considerable variation in IRB (Institutional Review Board) policies [22]. While some IRBs have specific policies, others do not. They ultimately recommended that in cases in which it was expected that more than five individuals with LEP would be recruited to a study, consent documents should be translated [22]. Although recognizing that the cost of doing so could be prohibitive, in their interpretation of US laws and guidelines, simply excluding individuals with LEP was deemed both illegal and unethical [22]. Laws and practices have continued to evolve in the US since. We did not identify clear guidelines within the Canadian literature concerning research ethics board guidance, and there was no obvious precedent for an approach within our institution. We met with the SickKids research ethics board (REB) to discuss study design. We recognized several common goals. We also learned that our institutional REB had been discussing how to address these challenges prior. We were aware that many in our institution had wondered about methods for conducting studies with individuals with LOE but had not done so due to lack of clarity around methodology. Thus an important learning in developing studies with marginalized populations include collaboration with REB to strategically overcome what can initially be institutional/process barriers. Through meaningful partnership, dialogue with shared goals and objectives, we can uphold the highest ethical standards while advancing health equity.

### Study execution details

A. Recruitment Procedures

Our recruitment process involves participants who are identified as primarily speaking a LOE being recruited and consented in the hospital before discharge. Families with LOE are identified by screening admission lists that note families requiring interpreters. Additionally, other families that may prefer not to use an interpreter but are identified as possibly having LOE may be approached and administered a one-question survey to determine if they self-identify their English language proficiency as less than “very well” [4]. Multiple approaches for identification and recruitment recognize the heterogeneity of populations and can enrich the study sample. Families are provided information about the study, and all questions are answered before formally seeking verbal consent with a pre-approved conversation outline. The primary medical care team is not involved in obtaining consent for the study to avoid influencing participants to participate. Consent is obtained with an interpreter who speaks the family’s preferred language. Before the research team member documents consent, potential participants are expected to demonstrate understanding by clearly outlining expectations of participation and their ability to withdraw from the study at any time without any consequences. This approach balances the rigour of informed consent while being flexible.

2 Interview Logistics:

Once consent is obtained, the approach we take is to arrange a mutually convenient time for the virtual semi-structured interview with a researcher and a medical interpreter. As our approach emphasizes family-centeredness we aim to maximize flexibility for family participation. A post-discharge interview allows for the family to participate at a convenient time and place for them. From a data perspective it also allows them to consider the admission in its entirety. Along the theme of family centeredness. interviews can be conducted during day or nighttime and on weekends, depending on family preference. Interviews are to be done virtually, but if other methods, such as phone, are easiest for the family, there are plans to accommodate this. As a small token of appreciation, we give families a gift card to acknowledge their time and generosity in sharing their experiences. It is essential to ensure that families are supported from a technical perspective and a safe and engaging interview environment is created. Early results from our process indicate that families greatly appreciate the opportunity to set the time of their interview “on their own schedule”, giving them more control of the process and appreciate having the opportunity to do them in their home environment, providing a futther measure of comfort.

3 Interpreter Use:

Several different methodologies and approaches have been utilized in studies employing interpreters. Cross-language interpretation is when an interpreter first receives raw information from a source language. Through a complex process of navigating, conceptualizing and understanding the data, the interpreter re-expresses the information in a second language, taking into account the specific nuances and context of this language [23–25]. An interpreter’s influence on research findings, in this case, can be very significant [23–25]. Different levels of autonomy can be provided to the interpreter, ranging from “verbatim” to “independent”; that is, interpreting word-for-word to situations versus the interpreter playing a leading role in the interactions [19–21]. In the case of more verbatim interpretation, techniques to optimize data collection and transmission include ensuring interpreters are provided with careful, specific instruction and debriefing with interview teams after the interviews [23, 24].

Translation procedures employed are variable and include verbatim transcription in the original/native language of the participants, followed by an analysis of this transcript and back translation into the “dominant language.” Studies can also involve exclusive transcription and analysis of interpreted components of the discussion in the dominant language [25, 26]. There are no standardized protocols, and various studies rely on having members of the research team who are experts in multiple languages or rely on using multiple transcripts and rounds of transcription/translation. [26, 27]

To optimally utilize interpreter services we designed the following methodology: Before the semi-structured interviews, the research team discusses expectations and role in the interview with the professional medical interpreter. The interpreter is instructed to provide verbatim interpretation throughout the interaction. At the end of the interview, the researcher debriefs with the interpreter to discuss any additional observations and any process/procedural issues or insights the interpreter can provide. We transcribe all components of the interview that are in English (the interviewer, the interpreter’s interpretation, and any English the family may speak during the interaction). This methodology allows us to feasibly interview families who speak any language we can secure an interpreter for. While other studies have focused on a limited number of languages and thus employed other interpretation methods, our practical approach ensures appropriate rigour while maximizing our ability to engage with a much wider range of patients and families, capturing the heterogeneity of this population.

4 Youth involvement

Due to several ethical and logistical challenges, youth have often been excluded from research, weakening research findings [28–32]. Though many researchers study questions that focus on child health and wellbeing, much of the information is gleaned from parents and caregivers. Children bring their lens of experience and have a wealth of knowledge and important perspectives to share. Qualitative research can be a useful method to understand child/youth perspectives. Child/youth participation in research has increased significantly in recent years with advancements such as applying a human and child rights framework to recruitment and participation, and advances in social theories around child agency [28–32].

Concerning assent, children need the clear option to refuse participation, withdraw freely and participate openly. Like adults, they require specific information on how to refuse or withdraw and should be offered ongoing check-ins about their desire to continue and how they want the data to be utilized [28–32]. Assent processes and forms and study materials should be “child/youth friendly” and conducive to comprehension according to participant age/development [28–32]. In some situations, verbal assent may be more appropriate than signing documents [28–32].

Respecting family preferences and cultural expectations, children who participate may do so by interviewing alone, with their caregivers or a combination of them. Children who demonstrate capacity) will be asked to provide assent to participate in the study. Children are notasked to sign assent forms. Children may participate in English or their preferred LOE.. Our interview guide has specific questions geared toward understanding the youth perspective.

5 Interview Guide:

Our interview questions are crafted with feedback and guidance from our patient partner and the RFAC. Our interview approach is to engage in a conversation with participants to understand the depth and nuance of their lived experience, ensure we develop rapport, then work to co-develop opportunities for improvement and explore solutions. The interview guide is iteratively updated with initial interviews informing future interviews as is standard in qualitative methodology.

Through our study design process we will be working to address these objectives (1) To understand the lived experience of families with LOE around the hospitalization of their child and (2) To understand the perspectives of patients and families with LOE on how the healthcare system can improve care during hospitalization.

## Discussion

Patient engagement in healthcare research has been referred to as the “next evolution in healthcare delivery.” [33] Ample arguments justify increased patient engagement in what has traditionally been a “paternalistic” approach to healthcare research [34]. More broadly, there has been growing awareness of the importance of the inclusion of historically marginalized populations in research [35, 36]. Through our participatory study, we hope to seek a better understanding of the lived experience of these patients. The goal of detailing the process undertaken to develop this study is to spark conversation around best practice in working with patients and families who use LOE for healthcare communication.. This work is important as we anticipate that this study's findings will support the development of a research and intervention agenda in Canada to improve health outcomes for patients and families with LOE.

A scoping review on patient engagement in research in 2018 found that despite methods to engage patients in health research increasing, evidence regarding the impact on specific patients and healthcare outcomes was still lacking [37]. A previous systematic review in 2014 noted that engagement is mainly done in the beginning of research and less commonly during execution and translation [38]. Snow and colleagues took a qualitative approach to review literature and conducting interviews with patients and health providers about engaging marginalized individuals in healthcare planning [39]. As motivation for their work, they noted that a universal approach to engagement did not meet the needs of those with diverse and marginalized backgrounds. Their analysis and subsequent proposed models for consideration and highlighted the importance of ensuring that engagement with marginalized populations is not reduced to a single defined process. They noted the importance of iterative engagement, with adaptations to the engagement models depending on unique circumstances and the individuals/populations of interest at the time. We must clearly communicate our methodologies in patient engagement and evaluate effectiveness.

There are some studies that have begun focusing more on the experience of marginalized patients including those who use LOE for healthcare communication. Our study aims to take a more holistic approach in probing around the overarching lived experience to gain further insight on the hospital experience impact. Lived experience probes deeper in not only exploring an individual’s experience and decisions but also considering the implications, knowledge gained in a first-hand manner. From a community-based participatory research perspective, it is important to recognize that gathering understandable meaning of experience from patients and families is critical. This approach is complementary and essential to research that probe more specific, narrow questions.

As initial learning points from our study design, we highlight the importance of establishing institutional policies to support research groups to include patients from diverse backgrounds in their studies. Adopting a common set of principles of research and collaboration like ours can advance overall efforts. Research teams require training concerning interpreter use and other aspects of the research process when working with patients and families who speakLOE. Partnerships must be built relative to study aims, orientation and design—not just with the initial planning. We must consider and plan for resources that support study involvement by individuals with LOE. Table 2 summarizes essential considerations when designing a study with patients and families with LOE.

**Table 2:** Opportunities and Challenges encountered in developing a study with patients and families who use LOE

Opportunities/challenges	Approach
Initial study development and planning	
Partnerships with stakeholder groups Language barriers Patient and family Engagement in study design Research ethics protocols	Collaborate with key stakeholder in your institution who oversee patient and family centered care and interpreter services Co-design methods, learn about additional partnership opportunities from these and community stakeholder groups Ensure ongoing dialogue and communication to maximize study success and address research interests and needs of our partners Study team should include patient engagement expert/consultation. Engage local Research Family Advisory Council (RFAC) for feedback during study development Compensate expert patient representative with lived experience to support study development, execution and analysis. We strongly recommend the involvement of patient partners on the study team Consider meeting with local Research Ethics Board (REB) to clarify and develop protocols that protect research participants, align with research ethics principles and are designed to empower recruitment of this traditionally excluded group
Study execution and logistics	
Recruitment procedures Data collection Interpreters Youth involvement	Ensure recruitment done with Interpreters. In-person recruitment by the study team needs to be built on a strong working relationship with the primary clinical team. Multiple methods for identification of potential participants are recommended. Consider most appropriate consent and assent methods Ensure a family-centered data collection process. Be flexible with time and format of interview Consider all potential methods for interpreter deployment during the study process. Balance rigour of interpretation/translation with engagement of different populations Children and youth should be involved in studies as participants when considering pediatric health questions. Work with families to optimize participation. Ensure recruitment, consent and participation process is appropriate for age

## Conclusion

Patient engagement in research is often discussed, but we have a significant opportunity to improve our engagement with marginalized populations. Given the importance of addressing the well-documented health disparities that differential social determinants contribute to, there is an urgent need to develop research protocols that truly engage families with LOE to address the health disparities they experience. Through our process, we seek to offer an approach and develop a protocol to conduct research that engages children and families with LOE to improve health outcomes. Establishing institutional partnerships and adopting a common set of research principles and collaborative framework underlie our approach and initial learnings, which we hope spark additional work in this area.

### Limitations

The goal of our dissemination of the process to develop this study, that incorporates community based participatory research, and is geared to recruit patients and families who use LOE for healthcare communication is to spark ongoing conversation around best practices. No empiric results are presented as we have not yet completed the study and are not yet able to report on the overall results from this study approach but look forward to ongoing discussion and reviews on best practices for engaging marginalized populations in participatory research.

## Data Availability

There are no results/additional data for this manuscript.

## Abbreviations

LEP: Limited English Proficiency
LOE: Language Other than English
GPIU: General Pediatric Inpatient Unit
CIECFCC: Centre for Innovation and Excellence in Child and Family-Centred Care
PIRN: Pediatric Inpatient Research Network
RFAC: Research Family Advisory Council
